# Current rehabilitation practices in intensive care units: a preliminary survey by the Japanese Society of Education for Physicians and Trainees in Intensive Care (JSEPTIC) Clinical Trial Group

**DOI:** 10.1186/s40560-016-0190-z

**Published:** 2016-10-28

**Authors:** Shunsuke Taito, Masamitsu Sanui, Hideto Yasuda, Nobuaki Shime, Alan Kawarai Lefor, Japanese Society of Education for Physicians and Trainees in Intensive Care (JSEPTIC) Clinical Trial Group

**Affiliations:** 1 Division of Rehabilitation, Department of Clinical Practice and Support, Hiroshima University Hospital, Hiroshima, Japan; 2 Department of Anesthesiology and Critical Care Medicine, Jichi Medical University Saitama Medical Center, 1-847 Amanuma, Omiya-ku, Saitama, Saitama 330-8503 Japan; 3 Department of Intensive Care Medicine, Kameda Medical Center, 929, Higashi-cho, Kamogawa, Chiba 296-8602 Japan; 4 Department of Emergency and Critical Care Medicine, Hiroshima University, 1-2-3, Kasumi, Minami-ku, Hiroshima, 734-8551 Japan; 5 Department of Surgery, Jichi Medical University, 3311-1 Yakushiji, Shimotsuke, Tochigi 329-0498 Japan; 6 Japanese Society of Education for Physicians and Trainees in Intensive Care, 3-3-11 Hongo, Bunkyo-ku, Tokyo, 113-0033 Japan

**Keywords:** Rehabilitation, Physical therapist, Intensive care unit, Rehabilitation protocol, Early mobilization, Questionnaire survey

## Abstract

We conducted an internet survey targeting healthcare providers in intensive care units (ICUs) in Japan and received 318 responses. Eighteen percent of respondents replied that full-time physical therapists (PTs) exist in their ICUs. Practicing sitting upright or sitting in a chair is frequently performed, while standing and walking are occasionally performed for patients undergoing mechanical ventilation. However, only 16 % of respondents use staged rehabilitation protocols. This preliminary survey suggests that full-time involvement of PTs in the ICU and introduction of rehabilitation protocols may not be common in Japanese ICUs.

## To the editor

In 2009, Schweickert et al. [1] first reported that early rehabilitation intervention significantly improved physical and mental function of critically ill patients. Since then, a substantial number of studies have shown the efficacy and safety of early rehabilitation and mobilization in the intensive care unit (ICU) [2, 3]. However, the criteria for determining the timing of initiation and suspension of rehabilitation in ICUs vary among studies, and the existence of standard rehabilitation protocols and the levels of physical therapist (PT) involvement for early mobilization are not yet ubiquitous, even in the USA [4]. It is not surprising that high-quality investigations have not yet been performed to describe the current practice of physical therapy in ICUs in Japan. Therefore, we conducted a preliminary internet survey investigating current practice patterns of physical therapy in Japanese ICUs.

Between January 13 and January 25 in 2016, anonymous questionnaires were distributed to physicians, nurses, PTs, occupational therapists (OTs), and speech therapists (STs), via the website of the Japanese Society of Education for Physicians and Trainees in Intensive Care (JSEPTIC) [5]. In this survey, rehabilitation was defined as any one of the following activities: passive exercise motion, neuromuscular electrical stimulation, respiratory muscle training, use of a cycle ergometer, practicing sitting upright or sitting in a chair, standing, and walking conducted for a patient admitted to an ICU.

**Fig. 1:**
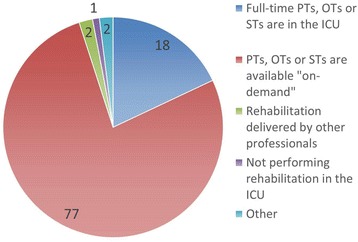
Involvement of therapists in rehabilitation in ICUs. *PTs* physical therapists, *OTs* occupational therapists, *STs* speech therapists

**Fig. 2:**
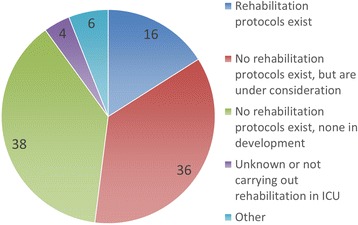
Existence of rehabilitation protocols

Of the 318 respondents, 39 (12.3 %) are doctors, 97 (30.5 %) are nurses, 175 (55.0 %) are PTs, OTs, or STs, and 7 (2.2 %) are clinical engineers or pharmacists. Institutional ICU staffing patterns of the respondents were categorized as closed ICUs (23 respondents, 7.2 %), ICUs with mandatory critical care consultation (102, 32.1 %), ICUs with elective critical care consultation (68, 21.4 %), ICUs with no critical care physician (110, 34.6 %), and unknown (15, 4.7 %). Fifty-seven respondents (17.9 %) responded that full-time PTs, OTs, or STs are involved in their ICUs, while 245 respondents (77.0 %) responded that they are involved “on-demand” (Fig. 1). Joint range of motion exercises, practicing sitting upright or sitting in a chair, and standing are frequently performed rehabilitation exercises for patients undergoing mechanical ventilation (Table 1). Respiratory muscle training and walking are occasionally performed. The majority of respondents answered that they never perform neuromuscular electrical stimulation or use a cycle ergometer. Despite the frequent conduct of sitting, standing, and walking in patients undergoing mechanical ventilation, only 51 respondents (16.0 %) replied that protocols for conducting staged rehabilitation for patients in the ICU exist (Fig. 2). Two hundred and thirty-six (74.2 %) answered that no such protocols exist, while approximately half indicated that protocols are currently under consideration. The decision to initiate rehabilitation in various patient conditions depends on the profession of the respondent and whether they are actively involved in rehabilitation practice or not (data not shown).

**Table 1:** Frequency of rehabilitation exercises performed for patients undergoing mechanical ventilation

	Frequently, *N* (%)	Occasionally, *N* (%)	Never, *N* (%)	Unknown, *N* (%)
Joint range of motion exercises	249 (78.3)	61 (19.2)	7 (2.2)	1 (0.3)
Sitting upright or sitting in a chair	207 (65.1)	91 (28.6)	16 (5.0)	4 (1.3)
Standing	134 (42.1)	134 (42.1)	41 (12.9)	9 (2.9)
Respiratory muscle training	105 (33.0)	155 (48.7)	46 (14.5)	12 (3.8)
Walking	63 (19.8)	135 (42.4)	111 (34.9)	9 (2.9)
Neuromuscular electrical stimulation	4 (1.3)	45 (14.2)	243 (76.4)	26 (8.1)
Cycle ergometer	7 (2.2)	42 (13.2)	248 (78.0)	21 (6.6)

Frequencies reported above may include multiple responders from a single institution

This preliminary web-based survey suggests that full-time involvement of PTs, OTs, or STs in the ICU and introduction of rehabilitation protocols may not be common in Japanese ICUs. In Europe, 75 % of ICUs had at least one PT working exclusively in the ICU [6]. Most ICUs in Australia also have at least one senior PT on staff [7]. This study suggests that rehabilitation may be provided “on-demand” in most ICUs in Japan. Recently, a consensus on active mobilization, including the criteria for determining when to start or suspend an intervention for patients undergoing mechanical ventilation, has been reported [8]. Risk stratification and safety standards regarding physical therapy and rehabilitation have also been reported [9]. However, this study suggests that in a majority of Japanese ICUs, rehabilitation practice may be performed on an individual-therapist basis, not following established protocols. It has been reported that following physical therapy protocols by an exclusively allocated PT decreased endotracheal intubation and reintubation rates in the ICU and hospital length of stay [10]. A study investigating the effect of protocolized rehabilitation by full-time PTs on patient outcomes is needed. This preliminary survey was made by voluntary participation of an individual respondent who viewed the website of the JSEPTIC Clinical Trial Group. The institutional information of individual respondents was not obtained, making it possible to have multiple respondents from one institution, not reflecting regional disparities in practice. It is acknowledged that selection bias of participants could result from an internet survey. Further studies are needed to clarify these limitations.

## Abbreviations

ICU: Intensive care unit
OTs: Occupational therapists
PTs: Physical therapists
STs: Speech therapists
